# Morphometric investigation of the infraorbital canal and determination of its “suspended” variant: a multidetector computed tomography study

**DOI:** 10.1016/j.bjorl.2026.101814

**Published:** 2026-04-11

**Authors:** Mehmet Selcuk, Hamza Kasar, Murat Inci, Nadire Unver Dogan, Selim Cınaroglu, Mehmet Ozturk

**Affiliations:** a Nigde Ömer Halisdemir University, Faculty of Medicine, Department of Anatomy, Nigde, Turkey; b Selcuk University, Konya, Turkey; c Karamanoglu Mehmetbey University, Ermenek Uysal and Hasan Kalan Vocational School of Health Services, First and Emergency Aid Program, Department of Medical Services and Techniques, Karaman, Turkey; d Selcuk University, Faculty of Medicine, Department of Anatomy, Konya, Turkey; e Selcuk University, Faculty of Medicine, Department of Radiology, Konya, Turkey

**Keywords:** Maxillary sinus, Infraorbital canal, Multidetector computed tomography, Haller cell

## Abstract

**Objectives:**

The aim of this Multidetector Computed Tomography (MDCT) study was to investigate the morphometry and variations of the Infraorbital Canal (IOC), including the “suspended” (Type 3) variant, to highlight its clinical importance in preventing iatrogenic nerve injury during sinonasal surgeries.

**Methods:**

In this retrospective analysis, 600 IOCs were analyzed using MDCT images obtained from 300 patients (18–82 years old, equal gender distribution). IOCs were classified into three types according to maxillary sinus protrusion: Type 1 (no protrusion), Type 2 (partial) and Type 3 (complete/suspended). The presence of Haller cell was also evaluated.

**Results:**

The prevalence of type 3 IOC was 13%. Haller cells were observed in 21.2%. Type 3 IOCs exhibited significantly larger D1 (distance from the infraorbital rim to the infraorbital foramen) and D2 (maximum length of the IOC) lengths and were found in younger patients. There was a weak negative correlation between age and D1/D2. Although women were older, IOC measurements did not show a significant gender difference in Type 3 cases, except that right-sided D3 (length of the bony septum extending into the maxillary sinus) was larger than left-sided D3.

**Conclusions:**

Type 3 IOC is a common anatomical variation, often associated with Haller cells, that increases the risk of infraorbital nerve injury in maxillary sinus surgery. Preoperative multidetector CT imaging of these variants is crucial to improve surgical safety.

**Level of evidence:**

5.

## Highlights

• MDCT was used to evaluate 600 infraorbital canals in 300 patients.
• Suspended (Type 3) canal prevalence 13%, linked to Haller cells in 21.2%.
• Type 3 canals were larger (D1, D2) and more common in younger patients.
• Type 3 IOC raises nerve injury risk in sinus and endoscopic surgeries.
• Preoperative MDCT is key to detect variants and improve surgical safety.

## Introduction

The Infraorbital Nerve (ION), the terminal and largest branch of the maxillary nerve, exits the skull base through the foramen rotundum, passes into the orbit via the inferior orbital fissure, and runs along the infraorbital groove before entering the Infraorbital Canal (IOC). It provides sensory innervation to the lower eyelid, conjunctiva, upper lip, and lateral nose.^1^, ^2^, ^3^, ^4^, ^5^, ^6^

Cranial nerve anomalies in the sinonasal region are critical for endoscopic surgeries. Variations in the sphenoid and maxillary sinuses usually arise from adjacent structures.^7^^,^^8^ The IOC, covered by a thin bony layer, is the weakest part of the orbital floor and contributes minimally to its structural integrity.^9^ Although the ION and artery are protected within the canal, they remain vulnerable to trauma during surgery.^10^

In some cases, the IOC separates from the orbital floor and protrudes into the maxillary sinus, increasing the risk of iatrogenic nerve injury as protrusion progresses.^3^^,^^11^ Such damage may cause hypoesthesia, paresthesia, or permanent neurological deficits.^4^^,^^6^^,^^12^^,^^13^ Therefore, detailed radiologic evaluation is essential before sinonasal surgeries.^6^^,^^12^^,^^14^^,^^15^ In implant surgery, sinus floor elevation may affect sinus drainage, making preoperative Computed Tomography (CT) crucial for assessing anatomical variations.^16^ However, radiologic data on the suspended Type 3 IOC remain scarce.^17^^,^^18^

Knowledge of the anatomical variations and morphometry of the IOC is critical for surgical safety. This study aims to comprehensively evaluate IOC variations, focusing particularly on the suspended Type 3 variant, and to emphasize its importance in clinical practice.

## Methods

### Study design

This study was approved by the Selçuk University Rectorate Local Ethics Committee (nº 2025/380, Date: 24 June 2025). The CT images used in the study were obtained retrospectively from the PACS (Picture Archiving and Communication Systems) archive of the Department of Radiology, Selçuk University. Images were acquired using a multi-detector CT scanner (Siemens Somatom Flash, Erlangen, Germany) with the following parameters 120 kV, 160 mA, rotation time 0.5 s, collimation 64 × 0.625, 220 mm FOV. Images were analyzed on a workstation (SyngoVia, Siemens, Germany). Power analysis was performed using G*Power 3.1.9.4, which indicated that at least 248 individuals were required (effect size = 0.46, confidence level = 95%, power = 95%).

Consecutive maxillofacial CT scans obtained between May 5, 2020, and June 15, 2025, were retrospectively reviewed. A total of 600 IOCs from 300 patients (150 males, 150 females) aged 18–82-years were evaluated. All images were analyzed in axial, coronal, and sagittal planes, and demographic data (age, gender, side) were recorded.

Patients with poor quality images, artifacts, previous maxillofacial trauma or intervention, or pathology in this region were excluded.

**Fig. 1:**
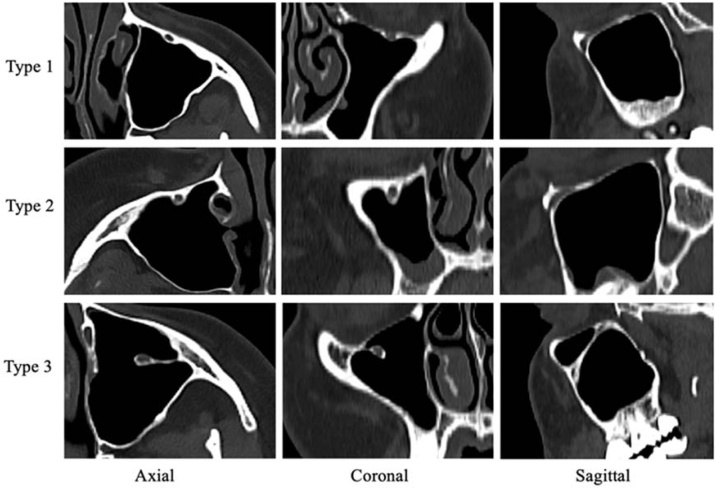
View of IOC types in axial, coronal and sagittal sections.

**Fig. 2:**
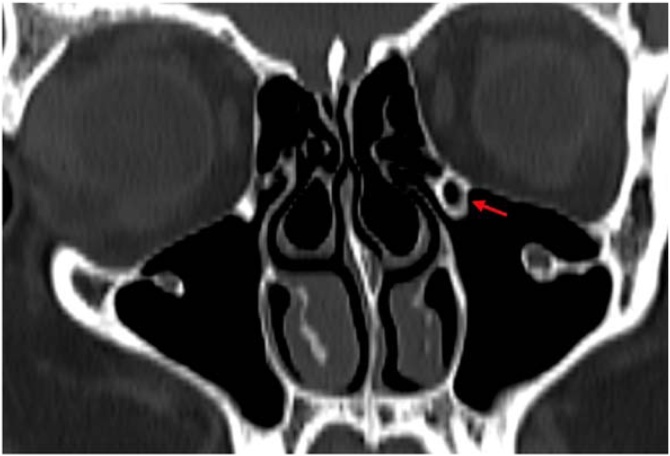
Haller cell (red arrow) in the coronal section.

All images were jointly evaluated by a radiologist and anatomist with 20-years of experience. The IOC was divided into three types based on its location in the maxillary sinus as described^1^ (Fig. 1). The presence of Haller cells in the coronal plane was also examined (Fig. 2).

Type 1: IOC is located completely within the roof of the maxillary sinus and does not protrude into the sinus lumen.

Type 2: The IOC is located below the roof of the maxillary sinus, but is adjacent to the roof. It partially protrudes into the sinus lumen.

Type 3: The IOC protrudes completely into the maxillary sinus or descends into the sinus lumen with septa and remains suspended.

### Morphometric measurements

**Fig. 3:**
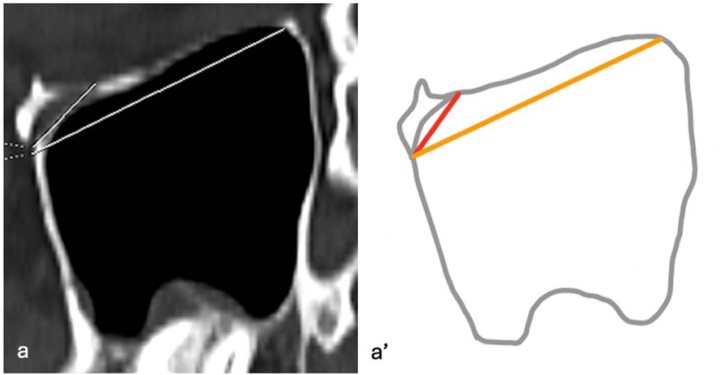
Distance from the infraorbital rim to the IOF (D1) and maximum length of the IOC (D2). (a) Sagittal image, D1 and D2 measurement; (a') Schematic drawing ‒ D1, red line; D2, orange line.

**Fig. 4:**
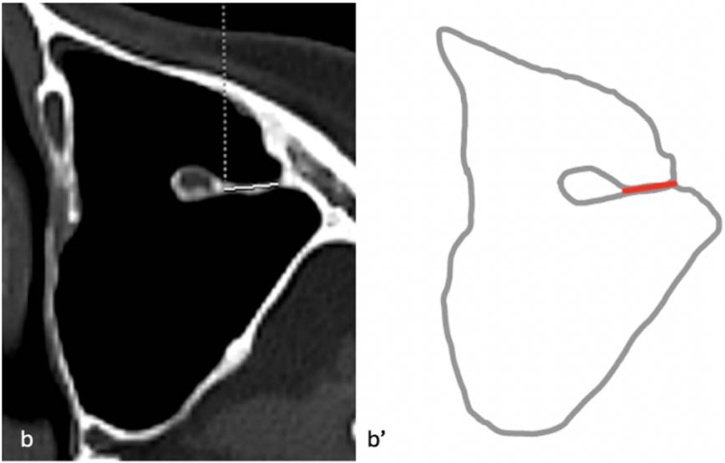
Maximum length of the bony septum from the canal to the maxillary sinus wall (D3). (b) Axial image, D3 measurement, (b') Schematic drawing ‒ D3, red line.

**Fig. 5:**
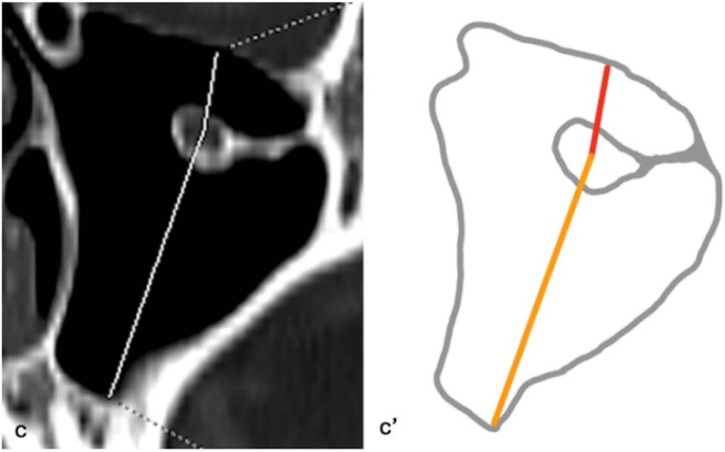
Vertical distance of the IOC to the sinus roof (D4) and maximum distance of the IOC to the sinus floor (D5). (c) Coronal image, D4 and D5 measurement; (c') Achematic drawing ‒ D4, red line; D5, orange line.

**Fig. 6:**
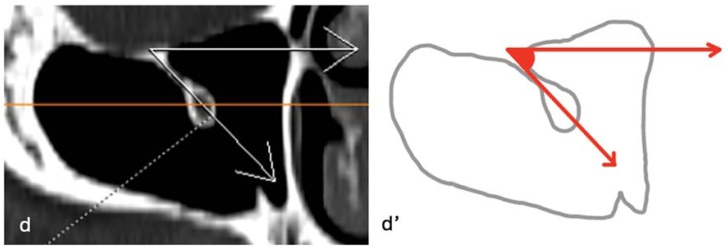
Angle (A1) between the reference line drawn parallel to the ground from the roof of the maxillary sinus and the second line drawn from the starting point of the IOC to the center of the canal. (d) Coronal image, A1 measurement; (d') Schematic drawing ‒ A1, between red arrows.

For all IOC variations, two basic measurements were performed in the sagittal plane: the distance from the infraorbital rim to the Infraorbital Foramen (IOF) (D1) and the maximum length of the IOC, defined as the distance from the most posterior infraorbital rim to the IOF (D2) (Fig. 3). In Type 3 IOC variations, four additional parameters were assessed: the length of the bony septum descending into the maxillary sinus (D3) on axial images (Fig. 4); the vertical distance of the IOC to the sinus roof (D4) and the maximum distance to the sinus floor (D5) on coronal images (Fig. 5); and the angle (A1) formed in the coronal plane between a reference line parallel to the maxillary sinus roof and a line drawn from the IOC origin to its canal center (Fig. 6). All measurements were performed bilaterally and evaluated separately.

### Statistical analysis

The data were analyzed using SPSS 22.0 (IBM Corp., Armonk, NY, USA). Variables with skewness and kurtosis values between −1.5 and +1.5 were considered normally distributed.^19^ Differences between genders were assessed using the Independent Samples *t*-test, while right/left side comparisons employed the Paired Samples *t*-test. IOC types were compared using one-way ANOVA with Post Hoc (Tamhane) tests. Chi-Square tests evaluated the distribution of Haller cells and IOC types by gender. Pearson correlation analysis was performed to examine the relationship between age and IOC lengths.

## Results

The mean age of the 300 patients (150 males and 150 females) was 36.72 ± 17.39 years (range: 18–82), with no significant age difference between sexes (p > 0.05). Among IOC variations, Type 3 was the least common (78 patients, 13%), Type 2 was observed in 204 patients (34%), and Type 1 was the most prevalent (318 patients, 53%). Haller cells were present in 127 patients (21.2%) and absent in 473 patients (78.8%). There were no significant gender differences in the distribution of IOC types or Haller cell presence on either side (p > 0.05) (Table 1).

**Table 1:** The prevalence of infraorbital canal types according to gender and side.

Canal type	All IOC	Right side			Left side		
		Female	Male	p	Female	Male	p
	n (%)	n (%)	n (%)		n (%)	n (%)	
Type 1	318 (53)	89 (59.3)	73 (48.6)	0.176	86 (57.3)	70 (46.6)	0.155
Type 2	204 (34)	41 (27.3)	53 (35.3)		47 (31.3)	62 (41.3)	
Type 3	78 (13)	20 (13.3)	24 (16)		17 (11.3)	18 (12)	
Total	600 (100)	150 (100)	150 (100)		150 (100)	150 (100)	
Haller cell	127(21.2)	33 (22)	35 (23.3)	0.445	29 (19.3)	30 (20)	0.885

No significant differences were observed between the right and left sides for D1 and D2 measurements (p > 0.05). Among 21 patients with bilateral Type 3 IOC, the right-sided D3 length was significantly greater than the left-sided D3 length (p < 0.05). However, no significant side differences were found for D4, D5, or A1 measurements in the same individuals (p > 0.05) (Table 2).

**Table 2:** Comparisons of distances (mm) according to side (mean ± standard deviation).

	n	Right side (mean ± SD)	Left side (mean ± SD)	p-value
D1 (mm)	300	9.91 ± 2.54	9.76 ± 2.32	0.238
D2 (mm)	300	28.51 ± 4.19	28.2 ± 4.07	0.255
D3 (mm)	21	4.82 ± 1.18	4.17 ± 1.02	0.005^a^
D4 (mm)	21	6.09 ± 2.08	5.85 ± 1.32	0.588
D5 (mm)	21	25.13 ± 5.76	23.85 ± 6.16	0.266
A1 (°)	21	56.57 ± 18.26	60.19 ± 16.54	0.470

a Paired Samples *t*-test (significance level p < 0.05).

Statistically significant differences were found between IOC types in terms of age, D1 and D2 parameters (p < 0.05). The mean age of individuals with Type 1 IOC was significantly higher than Type 2 and Type 3 groups (p < 0.05). D1 length was significantly different in all types, with Type 1 being the smallest and Type 3 being the largest, while D2 length was significantly smaller in Type 1 than in the other types (p < 0.05). In addition, the incidence of Haller cells was significantly lower in patients with Type 1 IOC than in patients with Type 3 IOC on both sides (p < 0.05) (Table 3).

**Table 3:** Comparison of IOC types.

	Age (years) (mean ± SD)	D1 (mm) (mean ± SD)	D2 (mm) (mean ± SD)	Haller cell (n)
Type 1 (n = 318)	40.25 ± 18.47	8.33 ± 1.25	27.09 ± 3.79	49
Type 2 (n = 204)	33.29 ± 15.19	10.62 ± 1.64	29.48 ± 3.85	40
Type 3 (n = 78)	31.32 ± 14.93	13.92 ± 2.15	30.57 ± 4.42	38
p-value	<0.001^a^	<0.001^a^	<0.001^a^	<0.001^b^

a ANOVA Test (significance level p < 0.05).

b Chi-Square Test (significance level p < 0.05).

The mean age of female subjects was statistically significantly higher than that of male subjects (p < 0.05). However, no significant difference was found between the genders in terms of morphometric measurements of the IOC (p > 0.05) (Table 4).

**Table 4:** Comparisons of the variables between males and females.

	All cases (mean ± SD)	Males (mean ± SD)	Females (mean ± SD)	p-value
Age (years)	36.72 ± 17.37	34.96 ± 15.99	38.49 ± 18.51	<0.001^a^
D1 (mm)	9.84 ± 2.43	10.06 ± 2.29	9.61 ± 2.55	0.063
D2 (mm)	28.36 ± 4.13	28.91 ± 4.06	27.80 ± 4.13	0.564
D3 (mm)	4.38 ± 1.21	4.62 ± 1.14	4.11 ± 1.24	0.863
D4 (mm)	5.56 ± 1.69	5.98 ± 1.81	5.09 ± 1.43	0.132
D5 (mm)	24.04 ± 5.92	26.1 ± 5.27	21.75 ± 5.82	0.400
A1 (°)	59.91 ± 15.62	57.61 ± 16.98	62.46 ± 13.75	0.227

Weak but statistically significant negative correlations were found between age and D1 and D2 measurements (age and D1 *r* = −0.250; age and D2 *r* = −0.254).

a Independet Samples *t*-test (significance level p < 0.05).

## Discussion

Accurate knowledge of IOC anatomical variations is critical for maxillofacial, orbital, and periocular surgeries, particularly in transmaxillary approaches such as Functional Endoscopic Sinus Surgery and Caldwell-Luc procedures.^6^^,^^12^^,^^20^ Type 3 IOC is located closer to the sinus roof or suspended in the sinus lumen, which significantly increases the risk of damage to the ION during surgical interventions.^1^^,^^9^ The 13% prevalence of Type 3 IOC in our study reveals that this variation is an important anatomical risk factor that should be considered in surgical planning. The literature emphasizes that if this variation is not adequately assessed before surgery, it may lead to misdirection of transmaxillary surgical corridors, difficulty in accessing tumors, and consequently, iatrogenic injury to the IOC.^20^

Previous studies reported that Type 3 IOCs position the Infraorbital Foramen (IOF) significantly below the orbital rim, an important anatomical feature to consider in Caldwell-Luc procedures.^1^ Similarly, the distance of the IOF from the orbital rim increases proportionally with the degree of IOC protrusion into the maxillary sinus, raising the risk of ION injury during transantral surgeries.^3^ In our study, the greater IOF-to-orbital rim distance in Type 3 IOCs aligns with these findings. However, the IOF-to-canine root distance remains constant across IOC types, suggesting that this landmark can serve as a reliable reference during surgery. Previous research also indicated that protruding IOCs are generally not close to the sinus floor, so the risk of iatrogenic injury during routine dentoalveolar procedures is low.^6^ Nonetheless, this risk increases in more invasive procedures involving the maxillary sinus, highlighting the importance of preoperative risk assessment based on anatomical variations and the surgical approach.

Considerable variation exists in IOC classification systems. Ference et al. described three types based on canal position relative to the sinus roof.1 Subsequent authors expanded this framework, with some subdividing Type 3 canals into categories based on septum length,^3^^,^^4^^,^^6^^,^^9^^,^^21^^,^^22^ while others proposed additional types, such as the lateroantral variant.^12^^,^^15^ Nam et al. further suggested a groove-based system.^23^ These inconsistencies complicate comparisons across studies and contribute to variability in reported prevalence, reinforcing the need for a standardized classification.

In the studies by Ference et al. and Açar et al., Type 1 was the most common, followed by Type 2, with Type 3 being the least frequent.^1^^,^^12^ These findings are consistent with our results but differ from those reported in other studies.^3^^,^^22^ We attribute these discrepancies to differences in sample size, ethnic or racial background, age distribution, imaging modalities used (CT vs. CBCT), and the classification criteria applied for defining IOC variations. The observed heterogeneity in the distribution of IOC types underscores the need for a standardized classification system (Table 5).

**Table 5:** Frequency of IOC Classifications in the Literature: A Comparison by Method and Study Type.

Researchers	Country	Method	n	Type 1 (%)	Type 2 (%)	Type 3 (%)	Type 4 (%)
Ference et al. (2015)	USA	CT	100	60.5	27	12.5	–
Yenigün et al. (2016)	Turkey	CT	750	12.3	51.2	36.4	–
Lantos et al. (2016)	USA	CT	500	–	–	10.8	–
Nam et al. (2017)	South Korea	Dry skulls and kadavra	20	12.5	65	22.5	–
Fontolliet et al. (2018)	France	CBCT	100	68.5	23.6	7.9	–
Haghnegahdar et al. (2018)	Iran	CBCT	192	26.5	50.3	23.2	–
Açar et al. (2018)	Turkey	CT	200	55.3	26.7	9.5	8.5
Kalabalık et al. (2020)	Turkey	CBCT	500	55.2	36	8.8	–
Li et al. (2020)	USA	Kadavra	10	30	60	10	–
Eiid and Mohamed (2022)	Egypt	CBCT	77	78.1	7.3	14.6	–
Karatag et al. (2025)	Turkey	CT	500	23.3	64.7	12	–
Our study	Turkey	CT	300	53	34	13	–

Ference et al. reported the mean distance between the infraorbital foramen and the orbital margin as 11.87 ± 2.46 mm, whereas Haghnegahdar et al. reported 9.39 ± 1.44 mm.^1^^,^^3^ The measurements reported by Haghnegahdar et al. are consistent with the D1 values in our study. Orhan et al. reported the maximum IOC length as 28.32 ± 3.85 mm on the right side and 27.46 ± 4.27 mm on the left side,^24^ which aligns with the D2 measurements obtained in our study. Overall, the increased D1 length observed in Type 3 IOCs reflects greater protrusion into the sinus lumen, representing a significant anatomical variation that may elevate the risk of nerve injury during surgical procedures.

In our study, four measurements (three lengths and one angle) were performed for Type 3 IOCs. The maximum length of the bony septum between the canal and the maxillary sinus wall (D3) was 4.38 ± 1.21 mm. Previous studies reported similar values: 4.9 mm by Gautam et al., 4 mm by Lantos et al., and 3.6 ± 2.1 mm (right) and 3.3 ± 1.5 mm (left) by Karatağ et al.^2^^,^^9^^,^^25^ The maximum vertical distance between the canal and the sinus roof (D4) was 5.56 ± 1.69 mm, compared to 8.58 ± 2.85 mm reported by Ference et al., 11.61 ± 2.55 mm by Haghnegahdar et al., and 6.76 ± 1.78 mm by Kalabalık et al.^1^^,^^3^^,^^6^ The maximum vertical distance to the sinus floor (D5) was 24.04 ± 5.92 mm, similar to 25.44 ± 4.69 mm reported by Kalabalık et al.^6^ In their study, D5 was significantly greater in men than women, which aligns with our findings. Additionally, we measured A1, an angle for which no direct comparison exists in previous IOC studies, providing novel data. Notably, Type 3 IOCs with higher angle values are located closer to the maxillary sinus midline, placing them at increased risk of iatrogenic injury during procedures such as Caldwell-Luc surgery, maxillary resections, sinus tumor removal, and endoscopic sinus surgery.

Previous studies have reported that individuals with Type 1 IOC are significantly older than those with Type 2 and Type 3 variants,^9^ which is consistent with our findings. Similarly, hyperplastic maxillary sinus (associated with Type 3 IOC) decreases with age, whereas hypoplastic maxillary sinus is more common in older patients.^12^ These observations suggest that age may influence IOC morphology and the frequency of its variants. In our study, no side differences were observed in D1 and D2 measurements, consistent with some studies but contrasting with others.^20^^,^^26^ Likewise, the prevalence of associated anatomical structures, such as Haller cells, showed no side differences.^6^^,^^15^ These discrepancies may reflect variations in population characteristics, measurement techniques, classification methods, or sample sizes. Clinically, detailed preoperative imaging of such variations is essential to reduce the risk of surgical complications.

Haller cells are important risk factors during surgery due to their proximity to the IOC.^9^^,^^27^^,^^28^ Previous studies reported the presence of Haller cells in Type 3 IOCs (27.7%), with this rate significantly increasing.^1^ Haller cells were found to be significantly more common in Type 2 and Type 3 compared to Type 1.^3^ In the same study, the prevalence of Type 3 IOCs increased from 14.8% to 29.1% in the presence of Haller cells and reached up to 43.9% when the nerve was located within the cell lamella. Likewise, other studies reported that the prevalence of Haller cells rises as the IOC type progresses.^9^ In our study, a statistically significant correlation was found between IOC types and the presence of Haller cells (p < 0.05); the lower-than-expected frequency in Type 1 and higher-than-expected frequency in Type 3 suggest that these cells may be associated with invasive Type 3 variants. On the other hand, some studies have found no significant relationship.^12^^,^^15^ It has been suggested that these inconsistencies may stem from differences in population, sample size, or classification.

A previous study examined the distribution of IOC types by gender and reported a correlation, with Type 1 being more frequent in women and Type 2 in men.^3^ In contrast, our study found no statistically significant association between gender and IOC types. These discrepancies may be attributed to ethnic, racial, or genetic anatomical variations among the populations studied.

Some studies reported no significant correlation between IOC length and age.^9^^,^^26^ However, in our study, statistically significant negative correlations were found between age and D1 and D2 measurements, suggesting a tendency for these dimensions of the IOC to shorten with increasing age. This relationship has been addressed in few studies and provides a novel contribution to understanding age-related anatomical changes. The observed differences may be related to variations in population demographics, age distribution, or the measurement and classification methods used.

### Limitations

Most studies on IOC variants focus on radiological analyses and lack direct clinical data on surgical outcomes. This situation makes it difficult to determine the true clinical significance of IOC variants or morphological features such as measured septum lengths, or the increase in the risk of iatrogenic injury. The studies also note that the rate of iatrogenic injury is currently unknown.

## Conclusion

Type 3 IOC is a common anatomical variation and is frequently seen in conjunction with other sinonasal variations such as Haller's cell. This condition increases the risk of ION injury, particularly in surgical procedures close to the maxillary sinus roof. Our study emphasizes the importance of careful preoperative evaluation of Type 3 variants protruding into the sinus using three-dimensional imaging (MDCT). In the future, prospective studies with larger samples from different populations and correlating radiological findings with surgical outcomes will strengthen the knowledge base in this field.

## ORCID ID

Mehmet Selcuk: 0000-0003-0798-1772

Hamza Kasar: 0000-0001-9635-920X

Murat Inci: 0000-0003-4933-3351

Nadire Unver Dogan: 0000-0001-5696-5547

Selim Cınaroglu: 0000-0002-4495-6106

Mehmet Ozturk: 0000-0001-5585-1476

## Authors' contributions

Conceptualization: Mehmet Selcuk, Murat İnci, Hamza Kasar, Nadire Unver Dogan, Selim Cınaroglu, Mehmet Ozturk. Methodology: Mehmet Selcuk, Hamza Kasar, Nadire Unver Dogan, Murat İnci, Mehmet Ozturk. Formal analysis and investigation: Mehmet Selcuk. Writing- original draft preparation: Mehmet Selcuk. Writing- review and editing: Hamza Kasar, Murat İnci, Nadire Unver Dogan, Selim Cınaroglu, Mehmet Ozturk. Supervision: Nadire Unver Dogan.

## Funding

This research did not receive any specific grant from funding agencies in the public, commercial, or not-for-profit sectors.

## Ethics approval statement

All procedures performed in this study involving human participants were in accordance with the ethical standards of the institutional and/or national research committee and with the 1964 Helsinki declaration and its later amendments or comparable ethical standards. Ethical approval (approval number 2025/380) was given by the Non-Intervention Clinical Research Ethics Committee of the Medical Faculty.

## Data availability statement

All data supporting the findings of this study are available upon request.

## Declaration of competing interest

The authors declare no conflicts of interest.
